# A Rare Case of Human Papillomavirus-associated High-grade Neuroendocrine Carcinoma of the Hypopharynx

**DOI:** 10.7759/cureus.4138

**Published:** 2019-02-26

**Authors:** Chandra Sanwal, Gerard Voorhees, Aaron Moon, Thomas Turner, Steven L Gates

**Affiliations:** 1 Internal Medicine, Corpus Christi Medical Center, Corpus Christi, USA; 2 Radiation Oncology, Corpus Christi Cancer Center, Corpus Christi, USA; 3 Radiology, Radiology Associates, Corpus Christi, USA; 4 Pathology, Corpus Christi Medical Center, Corpus Christi, USA; 5 Geriatrics, Corpus Christi Medical Center, Corpus Christi, USA

**Keywords:** small cell carcinoma, human papilloma virus, neuroendocrine carcinoma, hypopharynx, piriform sinus, human papillomavirus

## Abstract

We present a case of primary small cell carcinoma of the hypopharynx (SCCH), with a rare association with human papillomavirus (HPV). A comparison is made to 11 previously known, well-documented cases of SCCH with a review of the literature concerning SCCH. Our patient’s age of 23 years is the lowest among all previously reported cases, with an age range of 35-75. HPV association with SCCH is a rare new entity and eight HPV subtypes were identified in our case by ribonucleic acid (RNA) in-situ hybridization. The important common features among the previously reported 11 cases and our case include: (a) piriform sinus of the hypopharynx as the primary site in all cases, (b) a majority of patients presented with dysphagia and a neck mass, and (c) most patients had locoregional involvement at the time of presentation as opposed to distant metastasis. HPV-associated SCCH is extremely rare, with potentially aggressive clinical behavior, and needs much more research to further elucidate both the diagnostic and therapeutic approaches.

## Introduction

Small cell lung (SCC) neuroendocrine carcinomas (NECCs) are aggressive, with poor prognosis, and a mean survival of two to four months without treatment [1]. SCC in extrapulmonary sites (EPS) accounts for <2.5%-5% of all cases of SCC [1]. EPS are the esophagus, larynx, and bladder. The larynx is the most common site in the head and neck and SCC of the larynx accounts for <0.5% of all laryngeal carcinomas [1]. SCC of the hypopharynx (SCCH) is extremely rare. There are only 11 reported cases of SCCH to date [2]. The first case was reported in 1980 [2]. This case report presents the twelfth case of SCCH. We compare the characteristics of our case with the previously known 11 cases of SCCH with respect to rare new features and common characteristics reported in the previous cases. Our case is confirmed to be associated with eight different human papillomavirus (HPV) subtypes, which is a rare new entity. Among all the 12 reported cases of SCCH, we compare the age of diagnosis, anatomic location of the primary tumor, tobacco history, stage, histology, locoregional versus systemic treatment, metastases observed throughout the course of treatment, and follow-up. For our case, we also describe chemotherapy and the radiation therapy course along with follow-up imaging to monitor treatment response.

## Case presentation

**Figure 1:**
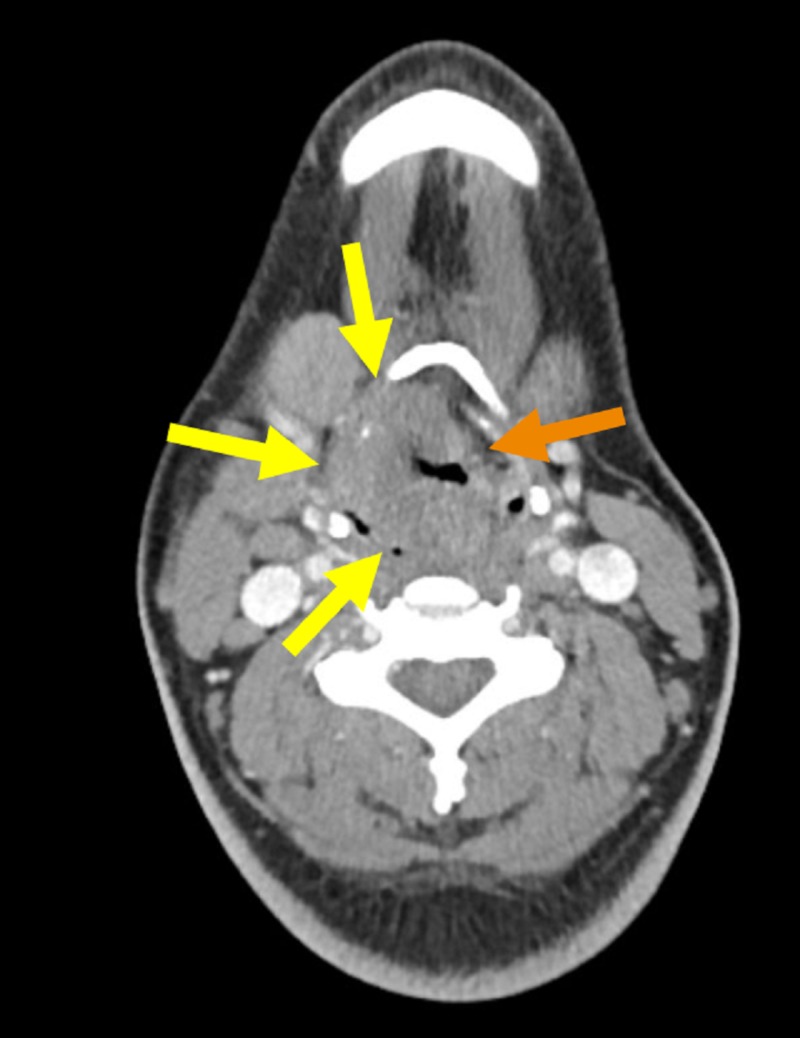
Axial contrast-enhanced computed tomography (CT) image of the neck. Yellow arrows indicate the mass (located within the right hypopharynx). Orange arrow signals the normal contralateral left hypopharyngeal wall.

**Figure 2:**
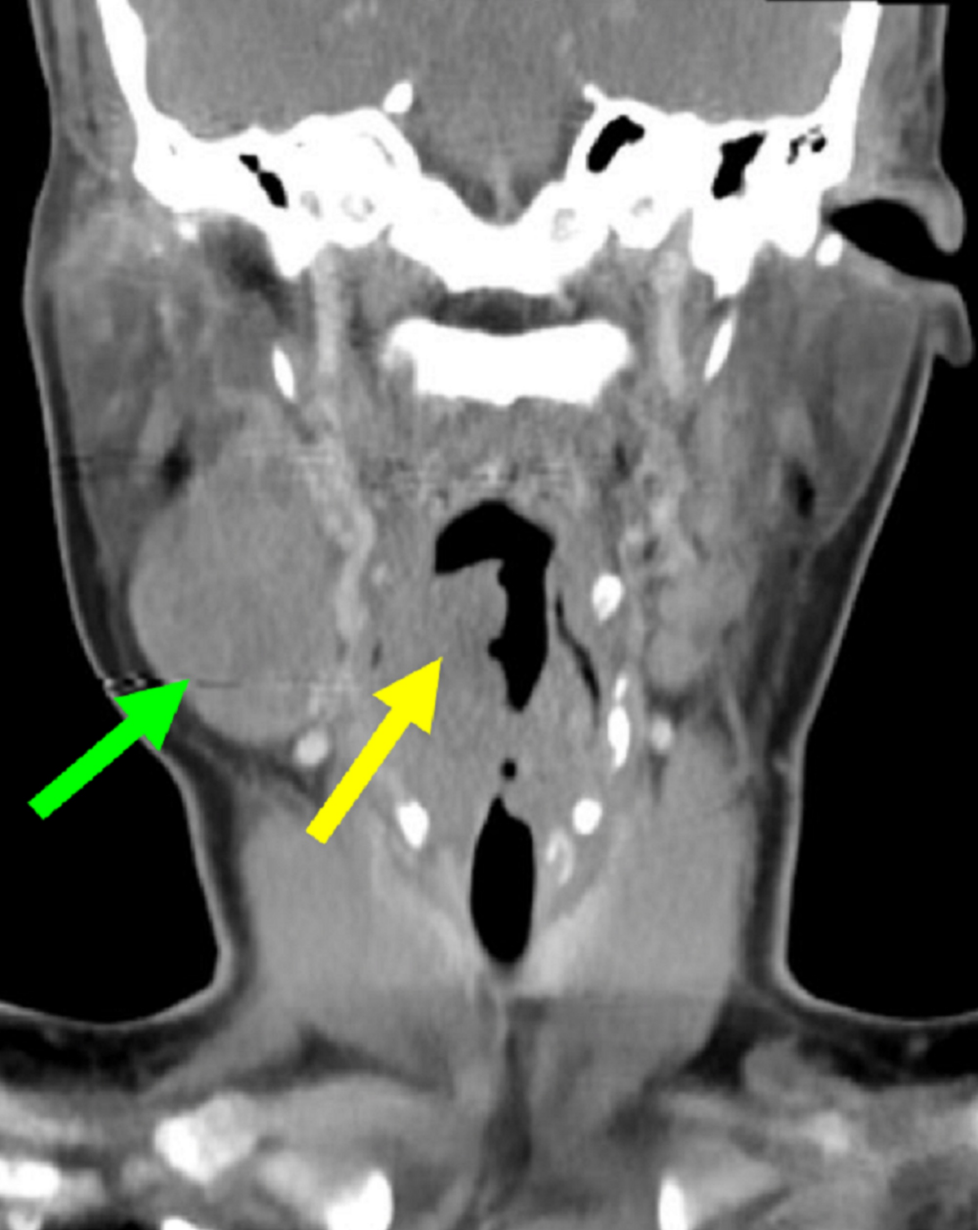
Coronal contrast-enhanced computed tomography (CT) image of the neck. Green arrow labels the right enlarged cervical lymph node. Yellow arrow indicates the primary hypopharyngeal mass.

We present a 23-year-old female with a past medical history of polycystic ovarian syndrome (diagnosed at the age of 15) and diabetes mellitus. She complained of persistent hoarseness, cough, and a decreased range of motion of her right neck, shoulder, and odynophagia. She rated her pain as six out of 10. Her pain was 100% relieved with tramadol. She lost 41 pounds in two months. A computed tomography (CT) scan of her neck showed a markedly enlarged, right level, 2/3 lymph node measuring 3.5x4.1x4.6 cm (Figures 1-2). There was also left level, 2/3 lymph nodes measuring up to 0.8x1.5 cm. The right aspect of the supraglottic larynx was asymmetrically thickened at 10 mm versus 3 mm on her contralateral left side.

**Figure 3:**
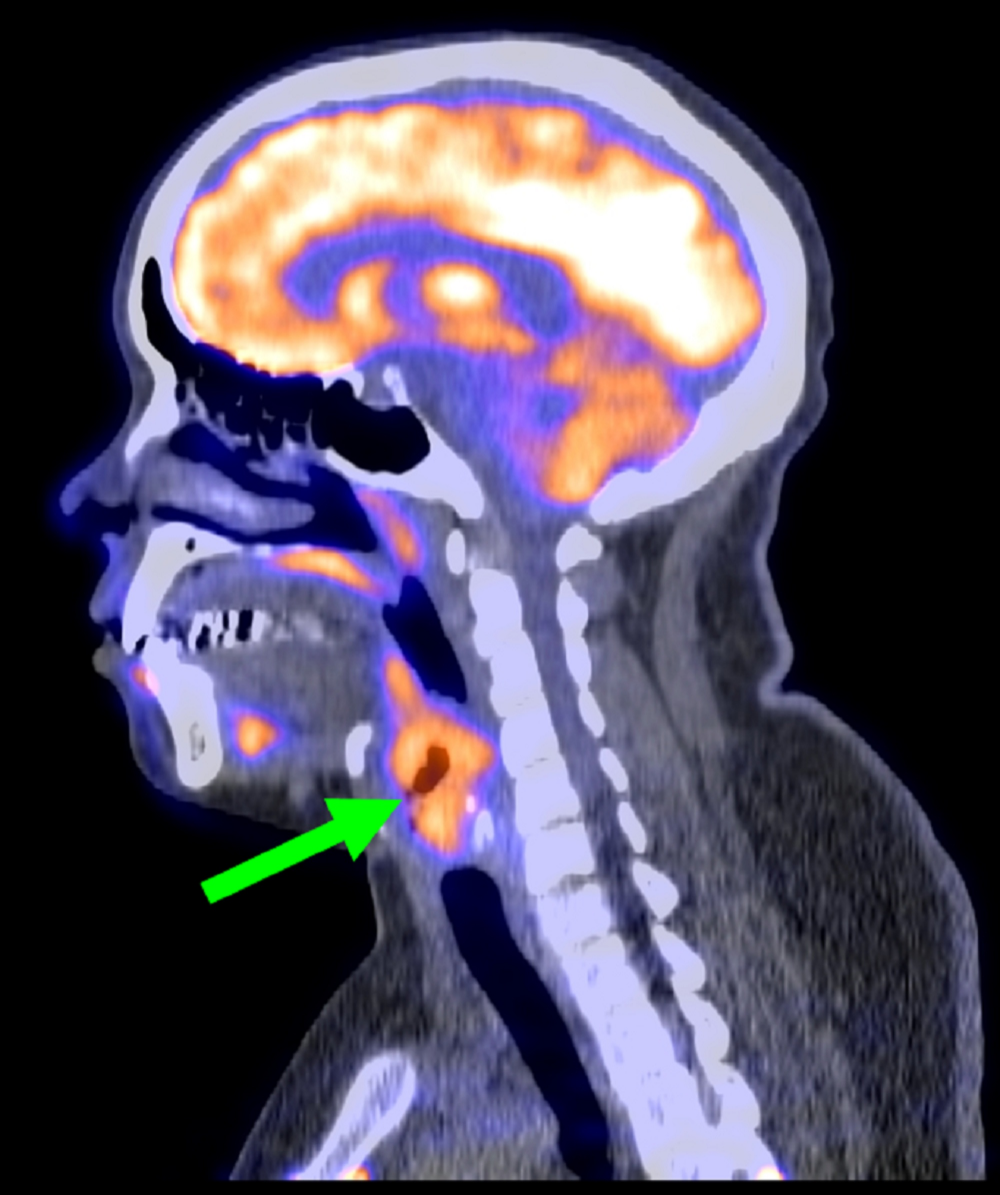
Sagittal positron emission tomography (PET) image of the neck. Green arrow labels a large, right level, 2-3 cervical node.

**Figure 4:**
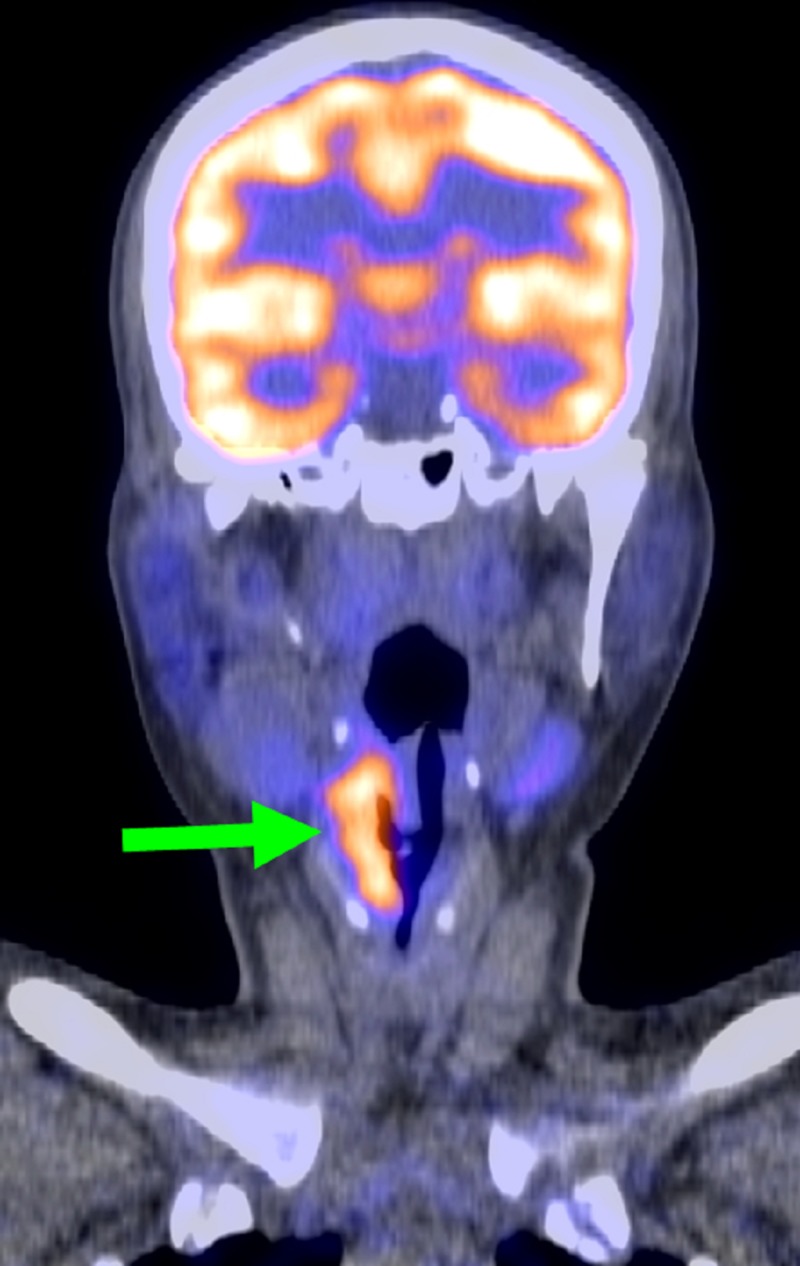
Coronal positron emission tomography (PET) image of the neck. Green arrow indicates the hypermetabolic, hypopharyngeal mass. This image is after the patient’s initial two cycles of chemotherapy. The metastatic cervical lymph node is no longer metabolically active.

The patient was evaluated by Ear, Nose, and Throat (ENT). Fiber-optic laryngoscopy showed her epiglottis was thickened and the right side was pushed to the left. She had a right pyriform mass with a fixed right true vocal cord and thickening of her right false vocal cord. Positron emission tomography (PET) scan showed a prominent, right-sided, hypopharyngeal, hypermetabolic mucosal mass consistent with a primary tumor involving her right vallecula, epiglottis, piriform sinus, and supraglottis (Figures 3-4).

**Figure 5:**
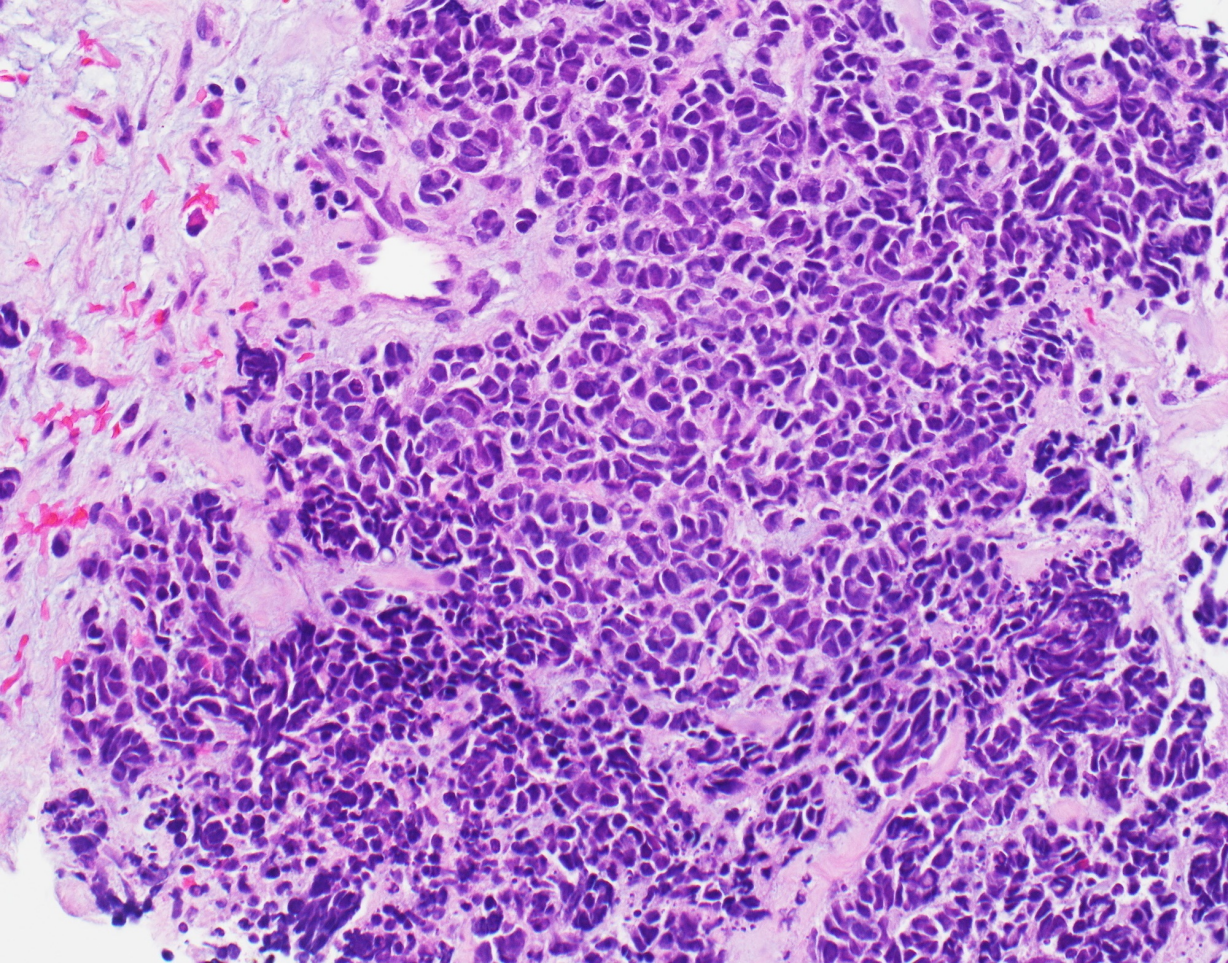
Histopathology of her neck mass shows small round blue cells. The cell borders are indistinct within the clusters of epithelium, with focal crush artifact and nuclear molding noted, characteristic of a poorly differentiated neuroendocrine neoplasm.

Ipsilateral hypermetabolic 4.9 cm level 2A and 3 hypermetabolic lymph nodes were seen. Subcarinal and left hilar hypermetabolic lymphadenopathy suspicious for nodal chest involvement was noted. An ultrasound-guided biopsy and fine needle aspiration (FNA) of the anterior cervical lymph node showed a small round cell tumor, favoring high-grade neuroendocrine carcinoma (Figure 5). A bone marrow biopsy showed normocellular bone marrow. No morphological or histochemical support for metastatic tumor was noted.

Pathology confirmed tumor expression of the p16 marker related to high-risk HPV (Figure 5). Eight HPV subtypes 16, 18, 31, 33, 35, 45, 52, and 58 were identified by ribonucleic acid (RNA) in-situ hybridization.

She received a total of six cycles of chemotherapy with cisplatin and etoposide. Two cycles were given neoadjuvantly, three were given concurrently with 70 Gy of localized radiation therapy, and the last cycle was given post-radiation therapy. After an initial two cycles of chemotherapy, a repeat PET scan showed a decrease in fluorodeoxyglucose (FDG) metabolism of the mass with no additional FDG-avid metastatic lesions. Resolution of the previously seen subcarinal and left hilar lymphadenopathy with no FDG uptake was also noted. After treatment, she had subjective improvement with increased neck range of motion, reduced odynophagia, and hoarseness.

## Discussion

Neuroendocrine (NECC) cancers have a combination of hormone-producing endocrine cells and nerve cells. Neuroendocrine cells are found throughout the body in organs such as the lungs and gastrointestinal tract, including the stomach and intestines. Neuroendocrine tumors can be found in all age groups, including children and young adults [3].

Small cell carcinoma (SCC) is one of many subtypes of NECC based on specific histologic and immunohistochemical properties [4-6]. SCC is most commonly found in the lung [1]. Extrapulmonary sites (EPS) are uncommon and account for only 2.5%-5% of all SCCs [1]. These most common EPS include the larynx, esophagus, and bladder [1]. The larynx is the most common EPS site in the head and neck and comprises <0.5% of all primary laryngeal carcinomas [1]. The hypopharynx has close proximity to the larynx and SCC of hypopharynx (SCCH) are extremely rare [1,7]. There are only 11 confirmed cases of SCCH to date [2]. This is the twelfth case of SCCH. This case report presents a comparison of SCCH with the previously reported 11 cases in terms of its common features and rare characteristics (Table 1).

**Table 1:** Comparison of clinicopathological features of 11 previous cases of SCCH and our case For more details please refer [2]. SCCH: Small Cell Carcinoma of Hypopharynx; PS: Piriform Sinus; LR: Locoregional; SCC: Small Cell Carcinoma; SQC: Squamous Cell Carcinoma; CRT: Chemoradiation Therapy; RT: Radiation Therapy; PCI: Prophylactic Cranial Irradiation; Sx: Surgery; Tx: Treatment

(#)Authors	Year/age/sex/smoking	Stage (TNM)	Main symptom	Site/extent of lesion at presentation		Histology	Locoregional Tx	Adjuvent Tx	PCI		Metastases (observed throughout the course)	Follow-up
(1) Ferlito and Polidoro	1980/57/M/+	T1N2bM0	Neck mass		PS/LR	SQC+SCC	Radical Sx + RT	None	−	Cervical nodes, bone, soft tissue		Died of cancer 3.5 months after diagnosis
(2) Mills et al.	1983/49/M/+	NA	Neck mass, dysphagia, weight loss		PS/LR	SQC+SCC	Radical Sx + RT	None	−	Cervical nodes		Alive free of cancer 6 months after treatment
(3) Baugh et al.	1985/63/F/−	T4a, more than N2c, M0	Dysphagia, cervical mass		PS/LR	SCC	Incomplete Sx	>4	−	Cervical nodes		Alive free of cancer 55 months after diagnosis
(4) Baugh et al.	1985/35/M/−	T3N2aM0	Dysphagia, weight loss		Base of tonsil to PS/LR	SCC	RT	None	−	Cervical node		Alive free of cancer 21 months after diagnosis
(5) Gaba et al.	2005/65/M/ex+	T4aN1M0	Dysphagia, weight loss		PS/LR	SCC	Incomplete Sx + CRT	>4	−	Cervical node		Alive free of cancer more than 24 months after diagnosis
(6) Sano et al.	2005/67/F/+	NA	Neck mass		PS/LR	SCC	RT	<4	−	Cervical node, lung, liver		Died of cancer 13 months after diagnosis
(7) Uwa et al.	2013/73/M/+	T4aN2bM0	Neck mass		PS/LR	SQC+SCC	Radical Sx	None	−	Cervical and mediastinum nodes, liver, lung		Died of cancer 9 months after treatment
(8) Bayram et al.	2015/50/M/+	T4aN2bM1 (lung)	Severe respiratory distress		PS/LR	SCC	Incomplete RT	<4	−	Cervical and lung		Alive free of cancer 15 months after treatment
(9) Misawa et al.	2016/74/M/+	T2N0M0	Throat pain, hoarseness		PS/Local	SQC+SCC	CRT	None	−	Bone		Died of cancer 7 months after treatment
(10) Nakahira et al.	2017/75/M/+	T2N2cM1 (bone)	Dysphagia		PS/LR-distant	SQC+SCC	None	>4	−	Bone, liver		Died of cancer 6 months after treatment
(11) Nakahira et al.	2017/73/M/−	T3N2bM0	Dysphagia		PS/LR	SQC+SCC	Radical Sx + RT	>4	+	Cervical nodes, lung		Alive with cancer 26 months after treatment
(12) Sanwal et al.	2018/23/F/Ex-smoker+	T3N3M0	Dysphagia		PS/LR		CRT	>4	-	Cervical nodes		Alive free of cancer 1 month after treatment

Our patient’s age of 23 years is the lowest among all previously reported cases, with an age range of 35-75. HPV associations with various types of NECCs of the head and neck have been reported. For instance, there are case reports of HPV-associated large cell NECCs of the head and neck and HPV-associated SCC of the oropharynx [8-10]. The association of HPV with SCC of the hypopharynx is a rare new entity. Pathology confirmed the tumor expression of the p16 marker related to high-risk HPV. Eight HPV subtypes 16, 18, 31, 33, 35, 45, 52, and 58 were identified by RNA in-situ hybridization. Some of the important common features among all the 12 cases (previously reported 11 and our case) include: (a) piriform sinus of the hypopharynx as the primary site in all cases; (b) a majority of patients presented with dysphagia and neck mass; and (c) most patients had locoregional involvement at the time of presentation as opposed to distant metastasis.

The therapeutic approach to NECC is guided by its proper characterization, whether it is pure large cell, pure squamous cell, pure small cell, or mixed type with or without HPV association [2,4]. HPV-associated SCC of the oropharynx is reported to have improved clinical outcomes [9].

SCC has a poor prognosis with a reported median survival of two to four months without treatment [1]. Surgery (total laryngectomy) alone does not improve local tumor control and decreases the quality of life [2,4]. Radiation therapy didn’t improve survival but it was successful in controlling the primary tumor site [4]. The combination of primary radiation therapy and adjuvant chemotherapy resulted in median survival of 55 months, representing a significantly longer overall survival than any other treatment regimens [4]. The recommended treatment is four to six cycles of etoposide plus platinum-based agents such as carboplatin and cisplatin [2]. As chemotherapeutic agents do not penetrate the blood-brain barrier, prophylactic cranial irradiation has been suggested as part of the management of this cancer [4]. Due to the very small number of reported cases of SCCH, the treatment guidelines are similar to that of SCC of the lung and there are no targeted therapies unique to SCCH [2]. Furthermore, HPV-associated SCCH is extremely rare with potentially aggressive clinical behavior and needs much more research to further elucidate both the diagnostic and therapeutic approaches.

## Conclusions

In all cases of SCCH reported to date, the primary site is found to be the piriform sinus of the hypopharynx. SCCH is a rare form of cancer, and unlike SCC of the lung, SCCH does not have any standard guidelines for its diagnosis and treatment. HPV-associated SCCH is even rarer. There is evidence that HPV-associated small cell of the oropharynx has improved clinical outcomes. It is yet to be researched how the association of HPV with SCCH impacts the disease prognosis and treatment. Hence, HPV-associated SCCH is a rare new entity with potentially aggressive clinical behavior and needs much more research to further elucidate its both diagnostic and therapeutic approaches.
